# PU.1-CD23 signaling mediates pulmonary innate immunity against *Aspergillus fumigatus* infection by driving inflammatory response

**DOI:** 10.1186/s12865-023-00539-2

**Published:** 2023-01-17

**Authors:** Min Wang, Ming Zhang, Jiayong Qiu, Chenyang Liu, Yao Lou, Tongsheng Wang, Yingmin Zhang, Yimin Mao

**Affiliations:** 1 Department of Respiratory and Critical Care Medicine, The First Affiliated Hospital, and College of Clinical Medicine, Henan University of Science and Technology, No. 24, Jinghua Road, Jianxi District, Luoyang City, Henan Province China; 2 Department of Respiratory Medicine, The Third Affiliated Hospital of Soochow University, Suzhou, China; 3 Department of Respiratory and Critical Care Medicine, The First Affiliated Hospital of Nanjing Medical University, Nanjing, China

**Keywords:** *Aspergillus fumigatus*, Innate immunity, THP-1-derived macrophages, CD23, PU.1

## Abstract

**Background:**

Aspergillosis is a common cause of morbidity and mortality in immunocompromised populations. PU.1 is critical for innate immunity against *Aspergillus fumigatus* (AF) in macrophages. However, the molecular mechanism underlying PU.1 mediating immunity against AF infection in human alveolar macrophages (AMs) is still unclear.

**Methods:**

In this study, we detected the expressions of PU.1, CD23, p-ERK, CCL20 and IL-8 and key inflammatory markers IL-1β, IL-6, TNF-α and IL-12 in human THP-1-derived macrophages (HTMs) or PU.1/CD23-overexpressed immunodeficient mice with AF infection. Moreover, we examined these expressions in PU.1-overexpressed/interfered HTMs. Additionally, we detected the phagocytosis of macrophages against AF infection with altered PU.1 expression. Dual luciferase, ChIP and EMSAs were performed to detect the interaction of PU.1 and CD23. And we invested the histological changes in mouse lung tissues transfected with PU.1/CD23-expressing adenoviruses in AF infection.

**Results:**

The results showed that the expressions of PU.1, CD23, p-ERK, CCL20, IL-8, IL-1β, IL-6, TNF-α and IL-12 increased significantly with AF infection, and PU.1 regulated the later 8 gene expressions in HTMs. Moreover, CD23 was directly activated by PU.1, and overexpression of CD23 in PU.1-interfered HTMs upregulated IL-1β, IL-6, TNF-α and IL-12 levels which were downregulated by PU.1 interference. PU.1 overexpression strengthened the phagocytosis of the HTMs against AF. And injection of PU.1/CD23-expressing adenoviruses attenuated pathological defects in immunodeficient mouse lung tissues with AF infection. Adenovirus (Ad)-PU.1 increased the CD23, p-ERK, CCL20, IL-8 levels.

**Conclusions:**

Our study concluded that PU.1-CD23 signaling mediates innate immunity against AF in lungs through regulating inflammatory response. Therefore, PU.1-CD23 may be a new anti-aspergillosis therapeutic for the treatment of invasive aspergillosis with the deepening of gene therapy and its wide application in the clinic.

**Supplementary Information:**

The online version contains supplementary material available at 10.1186/s12865-023-00539-2.

## Background

Invasive aspergillosis (IA) is a disease that seriously threatens human health. According to WHO statistics, the incidence of IA is increasing annually, with approximately 200,000 new IA patients worldwide each year, and the fatality rate is as high as 50%–95% [1, 2]. Although the development of antifungal drugs has greatly improved the prognosis of IA patients, the mortality rate of IA remains high due to the lack of rapid and reliable early diagnosis methods, the emergence of antifungal drug resistance and the limitation of treatment options [3]. The main pathogen of IA is *Aspergillus fumigatus* (AF), which accounts for approximately 90% of infections. AF conidia can enter the respiratory tract and alveoli through respiration. Recently, the role and mechanism of human innate immunity against fungal infections has received extensive attention, and it is of great significance to understand the pathogenesis of AF and guide clinical treatment to study immune recognition and the response to AF infection [4].

Alveolar macrophages (AMs) are the first sentinel of defence against pathogenic microorganisms. They eliminate invading AF through pattern-recognition receptor (PRR)-mediated endocytosis, release cytokines, recruit neutrophils and monocytes to the infection site, balance proinflammatory factors and anti-inflammatory factors, and coordinate the anti-*Aspergillus* response [5, 6]. There are four main types of PRRs: toll-like receptors (TLRs), C-type lectin receptors (CLRs), nod-like receptors (NLRs), and RIG-I like receptors (RLRs) [7]. CLRs account for the main PRRs recognizing fungal infections through extracellular C-type lectin domains (CRDs), which recognize important carbohydrate structures of most pathogenic fungi, such as β-glucan and mannan D [8, 9].

The haematopoietic transcription factor PU.1 belongs to the E26 transformation-specific (ETS) family and plays a broad range of roles in cell function, positively regulating gene expression in granulocytes, macrophages, B cells and dendritic cells [10–12]. Previous studies showed that PU.1 was critical for innate immunity against AF by regulating important CLR expression in human macrophages [13]. Moreover, it directly activated the dendritic cell-associated C-type lectin receptor (Dectin-1), enhancing the role of Dectin-1 in the host's innate immune response against AF [13]. It is well known that PU.1 activates downstream targets by binding to typical (A/G)AGGAAGTG motifs [12, 14, 15]. However, the role of PU.1 mediating innate immunity against AF infection in human AMs is still unclear.

Inflammation plays an essential role in the control of pathogens and in shaping the ensuing adaptive immune responses [16]. The differentiation antigen 23 (CD23) encoded by the Fc fragment of the IgE receptor II (FCER2) gene was recently considered to be a novel CLR [17, 18]. CD23 is a low-affinity IgE receptor and plays key roles in the IgE-mediated immune response, regulating cell differentiation and inflammation [17, 19, 20]. Phosphorylated-extracellular signal-regulated kinase (p-ERK)/ERK signaling regulates inflammatory response by enhancing the pro-inflammatory factors interleukin (IL)-1β, IL-6, TNF-α in UC patients [21]. Chemokine ligand 20 (CCL20) is an important circulating proinflammatory cytokines. Its expression is strongly associated with systemic inflammation [22]. Inflammatory factors involved in inflammatory disorders are important signatures of inflammation [23]. The pro-inflammatory cytokines IL-6, IL-1β, IL-8, TNF-α and IL-12 promptly produced in response to infections, promoting autoimmune inflammation [24].

In this study, the human THP-1 cells were induced into adherent THP-1 macrophages, mimicking human AMs. The expressions of PU.1, CD23, p-ERK, CCL20 and the critical inflammatory cytokines increased with AF infection in HTMs. And PU.1 regulated the expression of these genes. In addition, PU.1 directly activated CD23 transcription and overexpression of CD23 increased the levels of IL-1β, IL-6, TNF-α and IL-12 that decreased with PU.1 interference. PU.1-overexpressed human THP-1-derived macrophages (HTMs) promoted phagocytosis against AF conidia. Ad-PU.1 elevated the CD23, p-ERK, CCL20 and IL-8 expressions, and Ad-PU.1/CD23 alleviated histological defects in immunodeficient mouse lung tissues under AF conditions. Therefore, it indicated that PU.1-CD23 signaling pathway was critical regulator in anti-aspergillosis immunity by driving inflammation response, and may be a new anti-aspergillosis therapeutic for the future treatment of invasive aspergillosis.

## Methods

### Induced differentiation of HTMs

Human acute monocytic leukaemia mononuclear THP-1 cells (ATCC, TIB-202) cultivated in Dulbecco’s modified Eagle’s medium (DMEM) with 10% fetal bovine serum (FBS; Thermo Fisher Scientific, Waltham, MA, USA), 100 U/mL penicillin, and 100 mg/mL streptomycin (Thermo Fisher Scientific). The cell density was adjusted to 1.0 × 10^6^/ml. The human THP-1 cells were induced and differentiated into adherent THP-1 macrophages by phorbol-12-myristate-13-acetate (PMA, 100 ng/ml) stimulation for 24 h, mimicking human AMs.

### Plasmid construction and cell transfection

PU.1/CD23 overexpression constructions were established by cloning the full-length of PU.1/CD23 CDS to the empty pRK5-HA (Promega, US) vectors. The siRNA sequences against PU.1 were sense 5′-CAAGUAAAGUUAUUCUCAAUC-3′ and antisense 5′-UUGAGAAUAACUUUACUUGUU-3′. The HTMs were transiently transfected with CD23/PU.1 siRNA and pRK5-CD23 with Lipofectamine 2000 transfection reagent (Thermo Fisher Scientific, US) according to the manufacturer’s instructions controlled by a negative control.

### Observation of phagocytic ability of the macrophages

HTMs with PU.1 silencing and overexpression were incubated with FITC-labelled AFA1 (AF strains used in this study, referred to as AF) conidial suspensions (multiplicity of infection (MOI) = 1) for 4 h. The nuclei and cell membranes of the macrophages were stained with 4′,6-diamidino-2-phenylindole (DAPI) (Solarbio, C0065) and 1,1′-dioctadecyl-3,3,3′,3′-tetramethylindodicarbocyanine perchlorate (DiD) (Absin, abs47014947) fluorescent probes. A laser scanning confocal microscope (Lecia SP8, Germany) was used to observe the phagocytic ability of HTMs to AF.

### Establish of the AF infection model

HTMs were incubated with AF conidial suspensions (MOI = 1). Macrophages were harvested at 0 h, 8 h, 16 h and 24 h after AF conidial stimulation. Quantitative real-time polymerase chain reaction (qRT–PCR), Western blotting analysis and enzyme-linked immunosorbent assay (ELISA) were performed to quantify PU.1, CD23, p-ERK and critical inflammatory factor levels.

### qRT–PCR

Total RNA was extracted from HTMs with TRIzol reagent. The mRNA was reverse transcribed to cDNA and subjected to qRT–PCR with the PrimeScript^®^ RT Master Mix Perfect Real Time kit (TAKARA Bio Inc., Kusatsu, Japan) and SYBR Green Master Mix (Applied Biosystems, Foster City, CA, US). The reaction was conducted on a qPCR instrument (Applied Biosystems 7900HT Real-Time System, US). The relative gene expression levels were analysed by the 2^−ΔΔCt^ method and normalized to actin. The primer sequence information was shown in Table 1.

**Table 1:** The qRT-PCR primer sequence information

Gene	Upstream primer(5′-3′)	Downstream primer(5′-3′)
PU.1	GCACCTTCCAGTTCTCGTCCAAGC	CGCCGCTGAACTGGTAGGTGAGCT
CD23	ACTGCGTGATGATGCGGGGCTCC	GTCAG GGTCTGGTCTTGAATCAG
CCL20	TGATGTCAGTGCTGCTACTCC A	TACTGAGGAGACGCACAATATAT
ERK	TTCAGACATGAGAACATCAT	GCAGATATGGTCATTGCTGAG
IL-8	GAACTTAGATGTCAGTGCATAA	GACAGAGCTCTCTTCCATCAGA

### Western blotting analysis

The collected macrophages were lysed with RIPA protein extraction reagent (Beyotime, Beijing, China) supplemented with protease inhibitor cocktail (Roche, Pleasanton, CA, US). The lysis products were subjected to sodium dodecyl sulfate (SDS)-polyacrylamide gel electrophoresis (PAGE) gels for electrophoresis, transferred to polyvinylidene fluoride (PVDF) membranes, blocked in 5% milk, and incubated with primary antibodies (1:1000, rabbit anti-CD23, rabbit anti-PU.1, rabbit anti-p-ERK, rabbit anti-IL-8, rabbit anti-CCL20) (Abcam Inc., Cambridge, MA, US) and secondary antibodies (1:5000, goat anti-rabbit IgG) (Abcam Inc., Cambridge, MA, US). Autoradiograms were quantified by densitometry with GAPDH as a control (Additional file 1).

### ELISA

The inflammatory factor levels stimulated with AF infection in HTMs were detected using an ELISA kit (Solarbio, Beijing) according to the manufacturer’s instructions.

### PU.1/CD23-expressing adenovirus

Full length sequence synthesis of PU.1 and CD23 were developed by GenePharma (Suzhou, China). The sequence of PU.1 and CD23 were copied into the pAD-pIRES2-ERFP vector, respectively. The recombinant adenovirus plasmid pAD-PU.1-pIRES2-ERFP and pAD-CD23-pIRES2-ERFP were obtained and transfected into human embryonic kidney 293 (HEK293) cells (ATCC, CRL-1573) using Lipofectamine 2000 (Thermo, USA). HEK293 cells amplified the recombinant adenovirus AD-PU.1-ERFP and AD-CD23-ERFP (referred to as AD-PU.1 and AD-CD23) in large quantities. The recombinant adenovirus empty constructions served as a control.

### Animal experiment

To investigate the effect of PU.1 and CD23 in mouse lungs, we anaesthetized BALB/c mice with pentobarbital (70 mg/kg) and transfected 30 μl PU.1/CD23-expressing adenovirus AD-PU.1/CD23 (~ 3 × 10^8^ PFU) into the mice via trachea controlled by adenovirus expressing ERFP (Ad-ERFP). PU.1 and CD23 expressions in mouse lung tissues of each group were detected by qRT–PCR.

PU.1/CD23-upregulated BALB/c mice and the wild-types were injected intraperitoneally with cyclophosphamide (200 mg/kg) 1 d after adenovirus AD-PU.1/CD23 administration. The immunodeficiency mouse model was established after 4 d of continuous cyclophosphamide administration. Immunodeficient mice were intratracheally injected with AF conidia (5 × 10^6^ PFU) controlled by normal saline (NS) treatment. The mice were sacrificed with pentobarbital (70 mg/kg) 7 d after AF infection. The diseased lung tissue in mice was separated, and haematoxylin and eosin (HE) staining and immunochemistry analysis were performed to detect the pathological changes in the mouse lung tissues.

### Immunohistochemistry

Paraffin-embedded blocks were cut into 4-μm thick sections. The dewaxed and hydrated tissue sections were incubated with anti-PU.1 (Abcam, ab88082), anti-CD23 (Abcam, ab254162), anti-p-ERK (Abcam, ab229912), anti-CCL20 (Abcam, ab9829) and anti-IL-8 (Abcam, ab18672) antibodies for 2 h at room temperature and subsequently incubated with a goat-anti-rabbit antibody for 40 min. The degree of staining was determined with diaminobenzidine (DAB) chromogen (Bio-Rad, Inc., CA, USA) and detected with a microscope (Olympus, Japan).

### Luciferase assay

pRK5-PU.1 cells overexpressing PU.1 and pGL3-CD23 with the LUC reporter were constructed and transfected into HTMs with Lipofectamine 2000 transfection reagent (Thermo Fisher Scientific, US). Twenty-four hours after transfection, the HTMs were incubated with AF conidia for 12 h. The LUC signals were analysed by a dual luciferase reporter gene detection kit (Promega, US) and FlowJo V10 software (Ashland, USA).

### Chromatin immunoprecipitation assay (ChIP)

HTMs and AF conidia were co-incubated (MOI = 1) for 8 h. Then, formaldehyde (1%) was applied to crosslink the proteins and chromatin for 10 min at room temperature. After that, the macrophages were lysed with an Ultrasonic Breaker (Bioruptor, Belgium). Immunoprecipitation was performed with an IP-level PU.1 antibody (Abcam, US) and an EZ ChIP kit (Millipore, Germany). qPCR was performed to detect the binding activity of PU.1 to the promoter of CD23 after the immune precipitate was washed.

### Electrophoretic mobility shift assay (EMSA)

According to the ChIP results, biotin-labelled probes for different sites were designed. The nonbiotin-labelled probes were applied as competitive controls with 25-fold, 50-fold and 100-fold competition concentrations. Total proteins were extracted with a cytoplasm-nucleus protein extraction kit (KeyGEN BioTECH, Nanjing, China) following the instructions. The nucleoprotein and nucleic acid probes were combined and reacted. After electrophoresis, membrane transformation and ultraviolet crosslinking, chemiluminescence was used to detect the binding activity of PU.1 to CD23.

### Statistical analysis

SPSS 20.0 and SigmaPlot 12.0 were applied for statistical analysis. All data were represented as means ± SD. Independent group tests were performed using Student’s *t*-test and one-way ANOVA test. *P* < 0.05 was considered statistically significant.

## Results

### AF infection increased the expression of PU.1, CD23, p-ERK and pro-inflammatory factors CCL20, IL-8, IL-1β, IL-6, TNF-α, IL-12 in HTMs

**Fig. 1:**
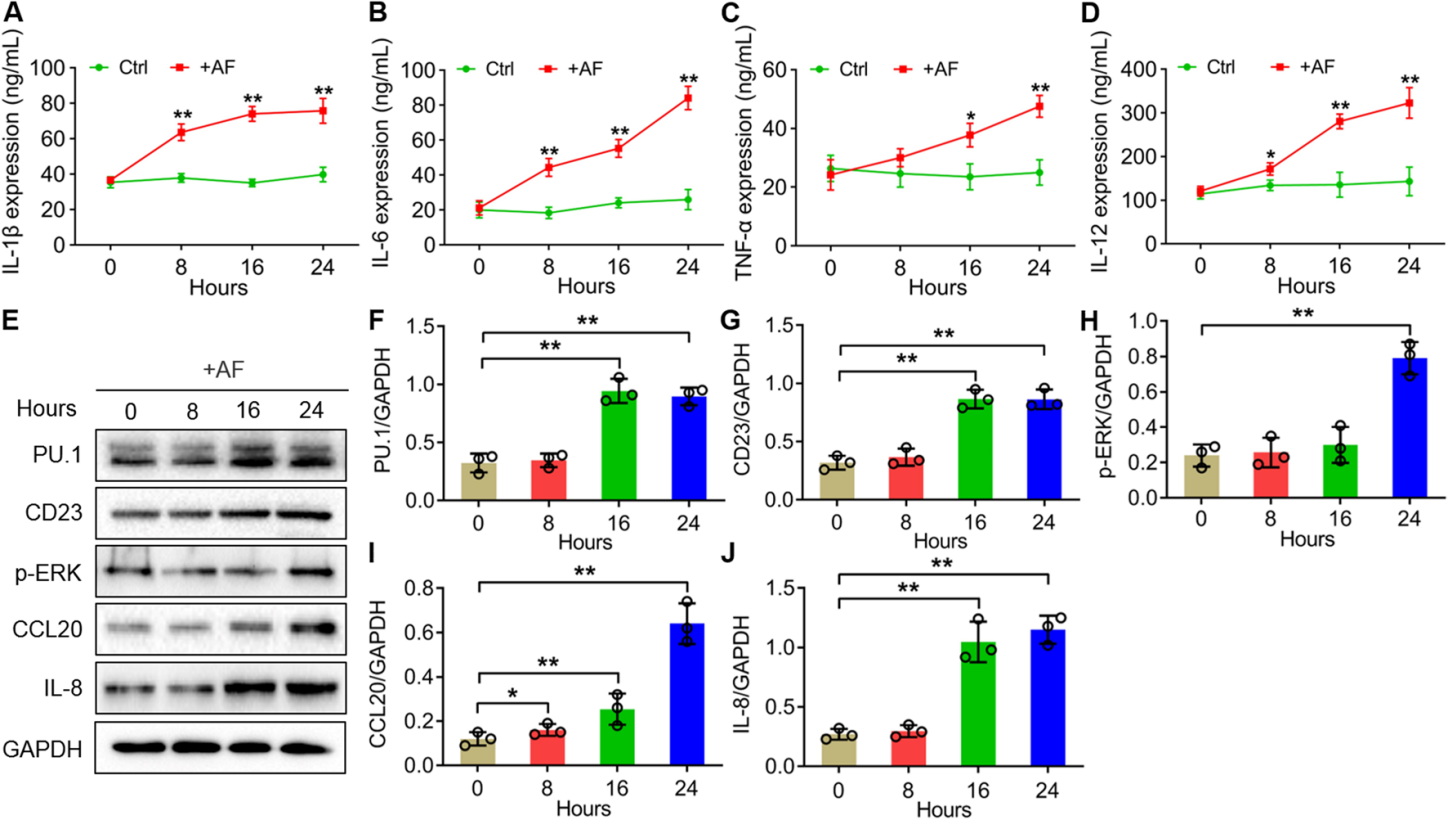
The protein expressions of IL-1β, IL-6, TNF-α, IL-10, PU.1, CD23, p-ERK, CCL20 and IL-8 in human THP-1-derived macrophages with AF infection within 24 h. **A-D** ELISA assay showed the levels of the inflammatory factors IL-1β (A), IL-6 (B), TNF-α (C) and IL-12 (D) in HTMs with AF infection. **E** Western blotting analysis showed the protein expressions of PU.1, CD23, p-ERK, CCL20 and IL-8 in HTMs with AF infection normalized to GAPDH. The samples derived from the same experiment and that gels/blots were processed in parallel. **F**–**J** Quantitative analysis of the expressions of PU.1, CD23, p-ERK, CCL20 and IL-8 in E. The X axis represents the infection time. All data are presented as the mean ± SD, N ≥ 3, *P < 0.05, **P < 0.01

To investigate the response of human AMs to AF infection, we induced human acute monocytic leukaemia mononuclear THP-1 cells to macrophages and infected with AF conidia (Fig. 1). Western blotting analysis showed that the protein levels of PU.1, CD23 and IL-8 were increased significantly since 16 h of infection compared to the controls, and after that these expressions basically remained at high level with no significant changes (**P < *0.01*) (Fig. 1E–G, J). And p-ERK expression was significantly increased at 24 h of infection (**P < *0.01*) (Fig. 1E, H). However, CCL20 was increased significantly since 8 h of infection, and continued to increase within 24 h (*P < 0.05, **P < 0.01) (Fig. 1E, I). According to the results from ELISA assay, the expressions of the inflammatory factors IL-1β, IL-6 and IL-12 in HTMs with AF infection increased significantly since 8 h, and TNF-α increased significantly since 16 h, when compared to the control groups (*P < 0.05, **P < 0.01) (Fig. 1A–D).

### PU.1 regulated the expressions of CD23, p-ERK, CCL20, IL-8, IL-1β, IL-6, TNF-α and IL-12 in HTMs

**Fig. 2:**
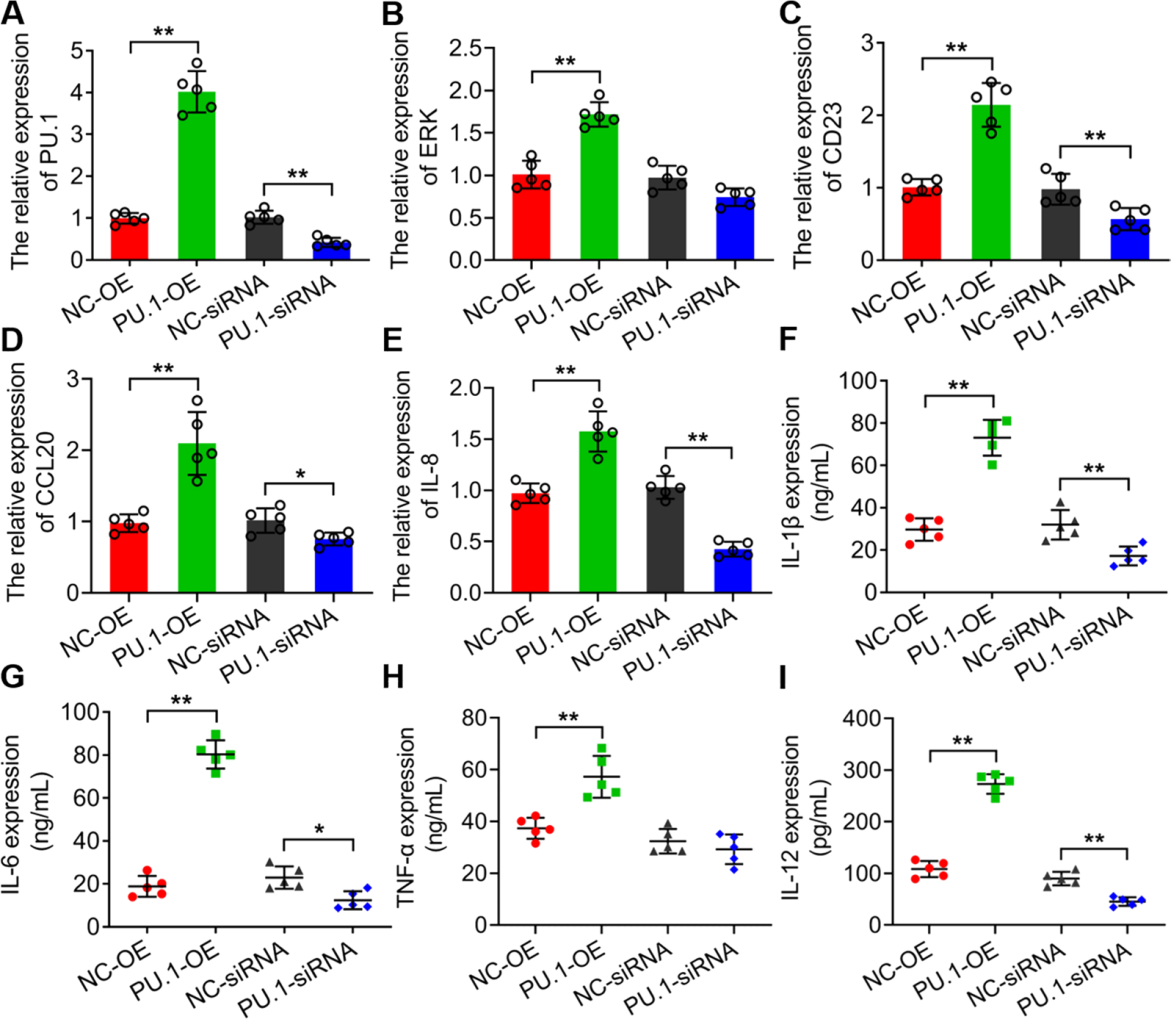
The expressions of PU.1 (**A**), ERK (**B**), CD23 (**C**), CCL20 (**D**), IL-8 (**E**), IL-1β (**F**), IL-6 (**G**), TNF-α (**H**) and IL-12 (**I**) in HTMs with PU.1 overexpression and silencing

To clarify the molecular function of PU.1 on these crucial factors in AF infection, we generated PU.1 overexpression and interference in HTMs. The qRT-PCR results showed that the mRNA expressions of PU.1, CD23, ERK, CCL20 and IL-8 were upregulated significantly with PU.1 overexpressed, but downregulated significantly with PU.1 interference except ERK (*P < 0.05, **P < 0.01) (Fig. 2A–E). Moreover, the ELISA assay revealed that the expressions of these pro-inflammation factors IL-1β, IL-6, TNF-α and IL-12 increased significantly with PU.1 overexpression, but reduced significantly with PU.1 interference except TNF-α (*P < 0.05, **P < 0.01) (Fig. 2F–I).

### PU.1 affected the phagocytosis of HTMs against AF conidia

**Fig. 3:**
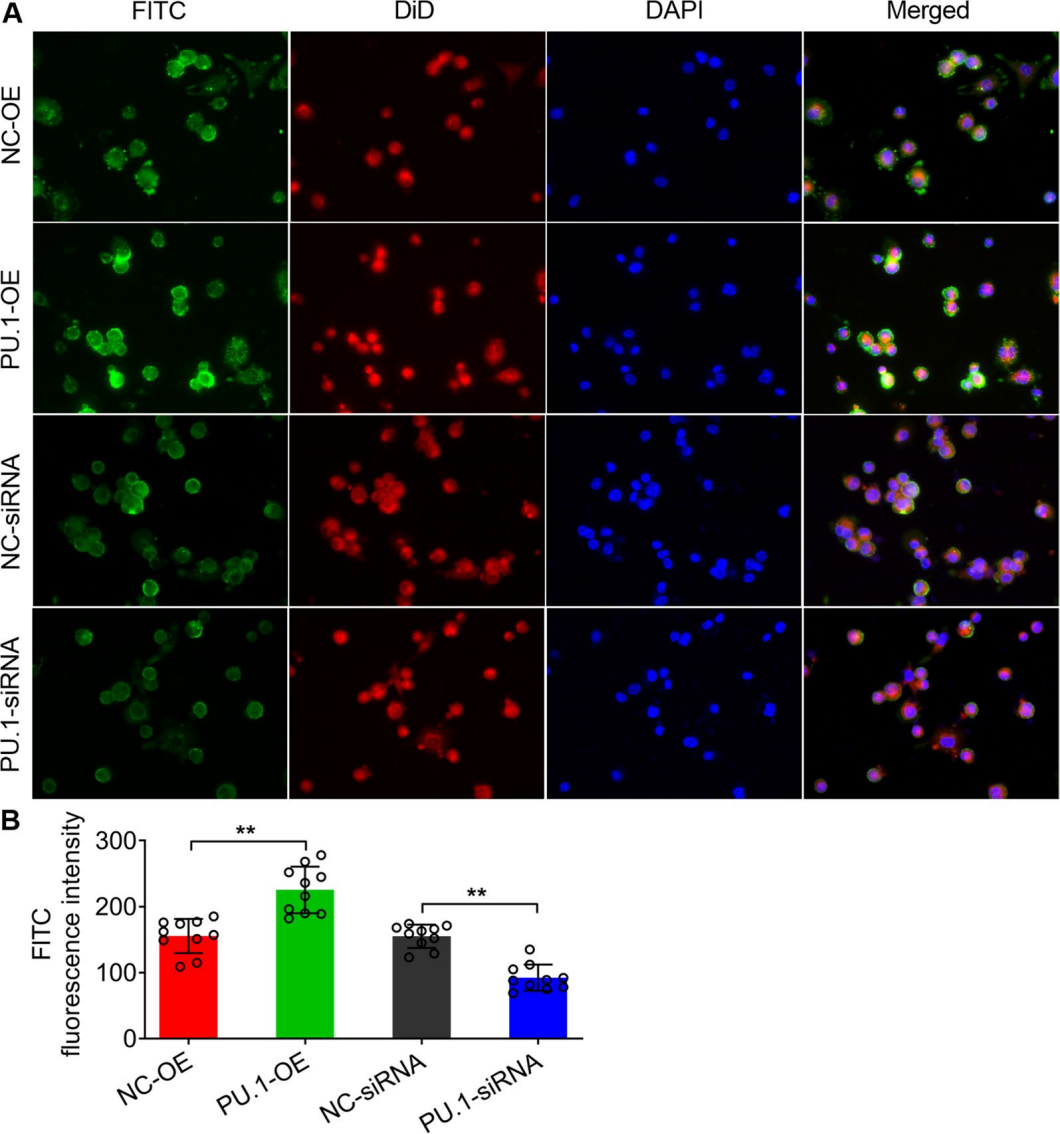
Phagocytosis of PU.1 overexpression and silencing. **A** Observation by confocal microscopy to detect the phagocytosis of HTMs against AF conidia with PU.1 overexpression and silencing. **B** The quantification of the FITC fluorescence intensity of A. All data are presented as the mean ± SD, N ≥ 3, **P < 0.01

To identify the role of PU.1 on the immune function of macrophages in response to AF infection, we established PU.1-overexpressed and silenced HTMs. After incubation of the PU.1-OE or PU.1 siRNA macrophages with FITC-labelled AF conidia, laser scanning confocal microscopy showed that PU.1-OE macrophages exhibited stronger phagocytosis against AF conidia than the control group (**P < 0.01) (Fig. 3A and B). In contrast, PU.1-siRNA macrophages revealed very weak phagocytic ability against AF conidia (**P < 0.01) (Fig. 3A and B).

### PU.1 directly activated the expression of CD23 in HTMs with AF infection

**Fig. 4:**
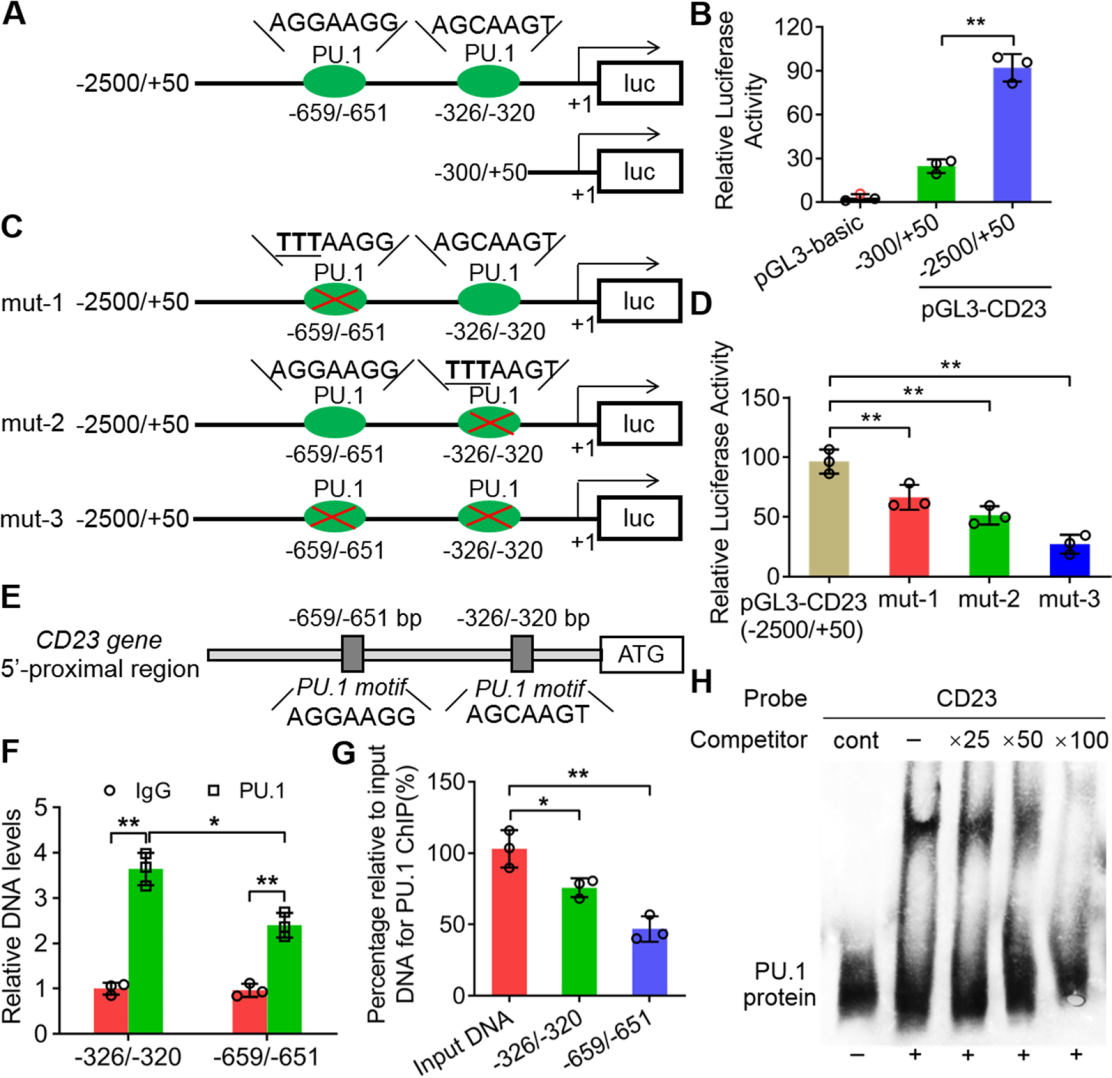
PU.1 directly activated the expression of CD23 in HTMs with AF infection. **A** Luciferase reporter plasmid containing two putative PU.1 binding sites in the CD23 promoter (2500 bp upstream of the ATG) compared to the full-length promoter deletion (300 bp upstream of the ATG). **B** Dual luciferase reporter assay showed that PU.1 could activate the LUC signal in the construct containing sequences from 2500 bp upstream of the ATG to 50 bp downstream of ATP. The luciferase activities were normalized to the β-galactosidase levels of the control. **C** Three artificial mutations in the PU.1 binding sites of the CD23 promoter. **D** Relative LUC activity showed the activating efficiency of PU.1 to the three artificial mutated CD23 promoters. **E** CD23 promoter revealed two putative PU.1 binding sites starting at -695 and -326. **F** Relative DNA levels of the CD23 promoter region containing two PU.1 binding sites from ChIP assay were detected by qPCR. **G** Percentage relative to input DNA for PU.1 ChIP was quantified. **H** EMSA showed that the PU.1 protein could bind to the CD23 promoter region in vitro. All data are presented as the mean ± SD, N ≥ 3, *P < 0.05, **P < 0.01

To characterize the downstream mediators of PU.1 in macrophages responding to AF infection, the bioinformatics analysis to the above genes regulated by PU.1 was carried out according to the reference [25], we found that the CD23 promoter region contained two putative motifs for PU.1 binding starting at − 659 and − 326 (Fig. 4A, E). The relative luciferase activity from the dual luciferase reporter assay showed that the PU.1 protein could activate the LUC reporter efficiently in the construct containing CD23 promoter sequences from 2500 bp upstream of ATG to 50 bp downstream of ATP (pGL3-CD23, − 2500/ + 50) compared to the basic plasmid (Fig. 4B). The deleted CD23 promoter containing − 300/ + 50 sequences revealed significantly weak relative luciferase activity (**P < 0.01) (Fig. 4B). Moreover, we mutated the two putative PU.1 binding motifs in turn to detect the contributions of the two motifs to the activation of the LUC reporter (Fig. 4C). The results showed that mut-1, mut-2 and mut-3 revealed significantly decreased relative luciferase activity compared to pGL3-CD23 (**P < 0.01) (Fig. 4D). Mut-3 with simultaneous mutation of two putative motifs led to a sharp decrease in the activation of LUC (**P < 0.01) (Fig. 4D). ChIP assays in AF infected THP-1-derived macrophages revealed that the PU.1 antibody significantly immunoprecipitated the two putative motifs in the CD23 promoter compared to the IgG controls (**P < 0.01) (Fig. 4E, F). The relative DNA levels containing the -326/-320 motif were even significantly higher than the ones containing the -659/-651 motif, which was consistent with the results from the dual luciferase reporter assay and percentage relative to input DNA (*P < *0.05,* **P < 0.01) (Fig. 4D–G). Furthermore, the EMSA verified the above results that the PU.1 protein could bind to the CD23 promoter region, which revealed an obvious mobility shift in electrophoresis via biotin labelling (Fig. 4H). The non-biotin-labelled probes contributed stronger competition activities as the amount increased (Fig. 4H).

### Overexpression of CD23 increased critical pro-inflammation factors’ expressions that were reduced by PU.1 interference under AF infection

Western blotting analysis showed that CD23 expression with PU.1 silencing decreased significantly under AF infection conditions (**P < 0.01) (Additional file 2: Fig. S1A, B). However, its expression increased significantly after overexpressing CD23 in PU.1 siRNA HTMs (**P < *0.01*) (Additional file 2: Fig. S1A, B). The ELISA assay showed the IL-1β, IL-6, TNF-α and IL-12 levels were significantly reduced in PU.1 interfered HTMs with AF infection compared to the NC-siRNA PU.1 group (**P < 0.01) (Additional file 2: Fig. S1C–F). However, overexpression of CD23 in PU.1 interfered HTMs significantly increased the expression of these inflammatory factors compared to the PU.1 siRNA + NC-OE CD23 groups under AF conditions (*P < 0.05*,* **P < 0.01) (Additional file 2: Fig. S1C–F).

### Exogenous PU.1 and CD23 alleviated pathological defects in immunodeficient mouse lung tissues with AF infection

**Fig. 5:**
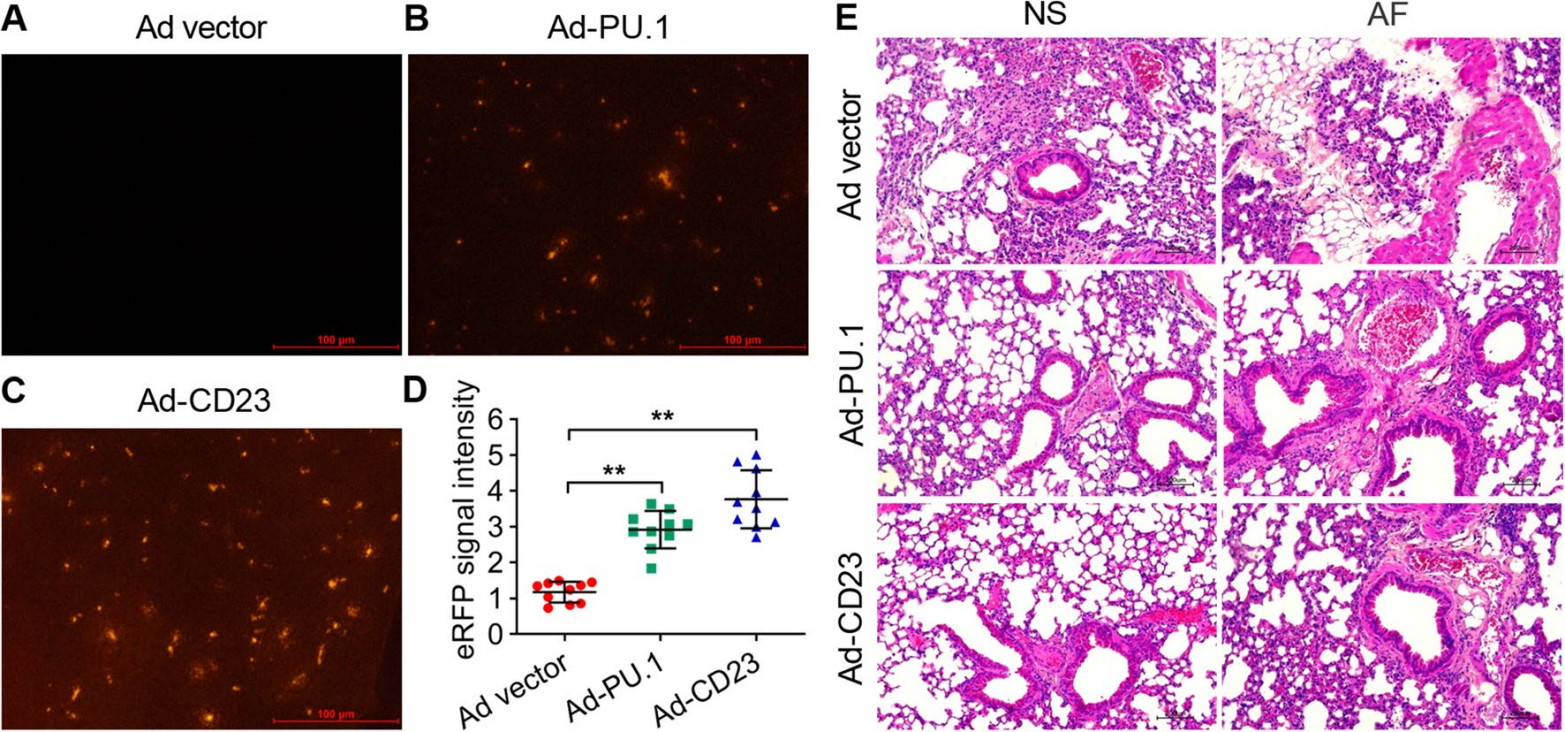
The histological changes from the mouse lung tissues with injection of PU.1/CD23-ERFP-expressing adenovirus (Ad-PU.1 and Ad-CD23 respectively). **A-C** ERFP signals detected in Ad-PU.1 and Ad-CD23 mouse lung tissues. **D** Relative levels of PU.1 and CD23 quantified by ERFP fluorescence. Adenovirus expression ERFP served as a control. **E** HE staining from mouse lung tissues injected with Ad-PU.1 and Ad-CD23 adenovirus in AF infection. Normal saline (NS) conditions and empty vector served as controls. All data are presented as the mean ± SD, N ≥ 3, **P < 0.01. Bars = 200 μm

To further figure out the biological function of PU.1-CD23 signaling in the immune response, we established PU.1- and CD23-high-expressing immunodeficient mice via intratracheal adenovirus transfection. The relative levels of PU.1 and CD23 were significantly increased following Ad-PU.1/CD23-ERFP adenovirus administration compared to the Ad-ERFP groups (**P < 0.01) (Fig. 5A–D). Moreover, the immunodeficient mice treated with empty Ad-ERFP vectors recruited massive leukocytes into the lungs from histopathological analysis after AF infection compared to the normal saline group (Fig. 5E). The alveoli and bronchioles were filled with macrophages, neutrophils, and lymphocytes, exhibiting symptoms of inflammation (Fig. 5E). However, the immunodeficient mice with high PU.1/CD23 expression revealed obviously milder inflammatory lesions in lung tissues (Fig. 5E). These results indicated that the exogenous upregulation of PU.1 and CD23 obviously alleviated pathological defects with AF infection in immunodeficient mouse lung tissues.

**Fig. 6:**
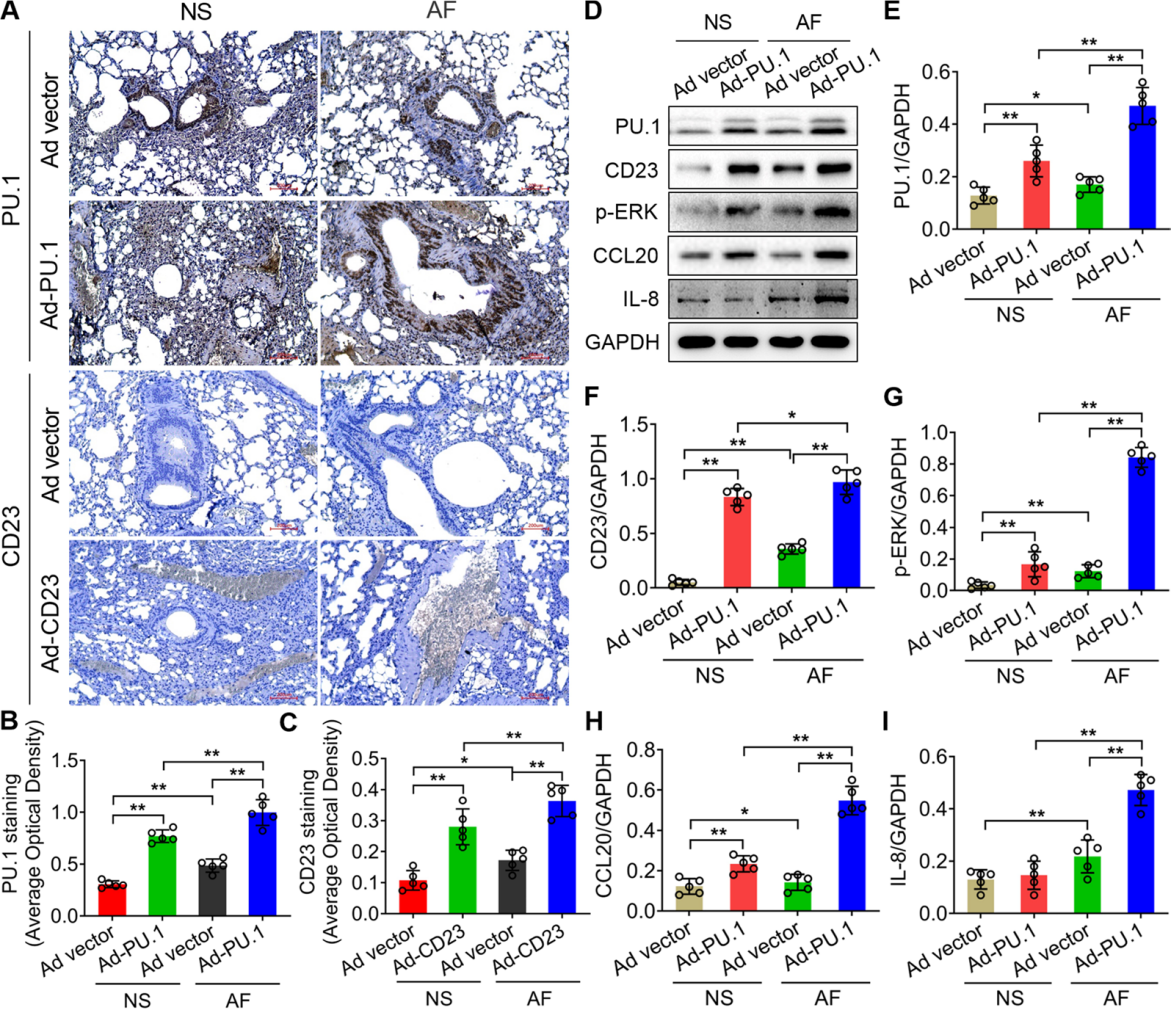
The expressions of PU.1, CD23, p-ERK, CCL20 and IL-8 in Ad-PU.1/Ad-CD23 mouse lung tissues under AF infecton and NS treatment. **A** Immunochemistry results showed the protein expressions of PU.1 and CD23 in mouse lung tissues injected with Ad-PU.1 and Ad-CD23 adenovirus with AF infection compared to the NS conditions. **B**, **C** The quantitative analysis of PU.1 and CD23 expressions in A. NS conditions and empty vector served as controls. **D** The western blotting analysis showed the protein levels of PU.1, CD23, p-ERK, CCL20 and IL-8 in mouse lung tissues injected with Ad-PU.1 adenovirus with AF infection compared to the NS treatment. The samples derived from the same experiment and that gels/blots were processed in parallel. **E**–**I** The quantification of protein levels in D. All data are presented as the mean ± SD, N ≥ 3, *P < 0.05, **P < 0.01. Bars = 200 μm

The results from immunochemistry revealed significantly higher PU.1 expressions in Ad-PU.1 immunodeficient mice compared to the empty vector group both under normal saline or AF infection conditions, and PU.1 levels of Ad-PU.1 mice were even significantly higher in AF conditions than the normal saline treatment (**P < 0.01) (Fig. 6A, B). Results about CD23 expression in Ad-CD23 mice showed similar changes when treated with normal saline or AF infection (*P < 0.05, **P < 0.01) (Fig. 6A, C). Furthermore, the western blotting analysis showed that the expression of PU.1, CD23, p-ERK, and CCL20 were upregulated in Ad-PU.1 mice compared to the empty controls both under normal saline and AF conditions (**P < 0.01) (Fig. 6D–H). But IL-8 expression was only upregulated with AF treatment but not in the normal saline treatment (**P < 0.01) (Fig. 6D and I). In addition, their expressions were upregulated under AF treatment compared with normal saline conditions (*P < 0.05, **P < 0.01) (Fig. 6D–I).

## Discussion

AMs are highly abundant in the lungs, contributing to homeostasis and immunity, and their functions are closely related to lung infections and chronic inflammatory disease [26]. According to the results in this study, PU.1 expression significantly increased in HTMs with AF infection, and PU.1-overexpressed HTMs strengthened but PU.1-interfered HTMs weakened the phagocytosis against AF conidia. Phagocytosis is a fundamental process in immunity in which AMs recognize and remove pathogens and particulates entering the airway with respiration through phagocytosis to defend against pathogenic microbial infections and maintain the balance and stability of the body's environment [27, 28]. And the phagocytosis by macrophages is an indispensable process to resolution of inflammation [29]. Thus, PU.1 upregulation might be beneficial to protect against AF infections by AMs.

AF as an opportunistic pathogen increases mortality in immunocompromised individuals, accounting for 90% of the IA incidence and 50–95% mortality rate [30–32]. The immune response is oriented by the interaction of pathogen-associated molecular patterns (PAMPs) in the cell wall and different PRRs from the host, leading to disease progression [33]. CD23 was suggested to be a fungal PRR sensing α-mannan and β-glucan in the AF cell wall [34]. Previous studies have indicated that CD23 expression is increased in AF keratitis in response to fungal infections [35]. In this study, the expression of CD23 was upregulated in HTMs with AF infection. It suggested that CD23 positively responds to AF infection in human AMs, which confirmed the previous finding that CD23 functions as a fungal PRR in antifungal immunity. Moreover, CD23 was directly activated by PU.1 based on dual luciferase, ChIP assay and EMSA assay. PU.1 is an important member regulating the activity of immune cells and served critical roles protecting against AF infection [36–38]. Taking together with PU.1 expression increased in HTMs with AF infection and PU.1 overexpression promoted the phagocytosis against AF conidia from macrophages, it indicated that PU.1-CD23 signaling plays a key role in innate immune response to AF infection in HTMs.

**Fig. 7:**
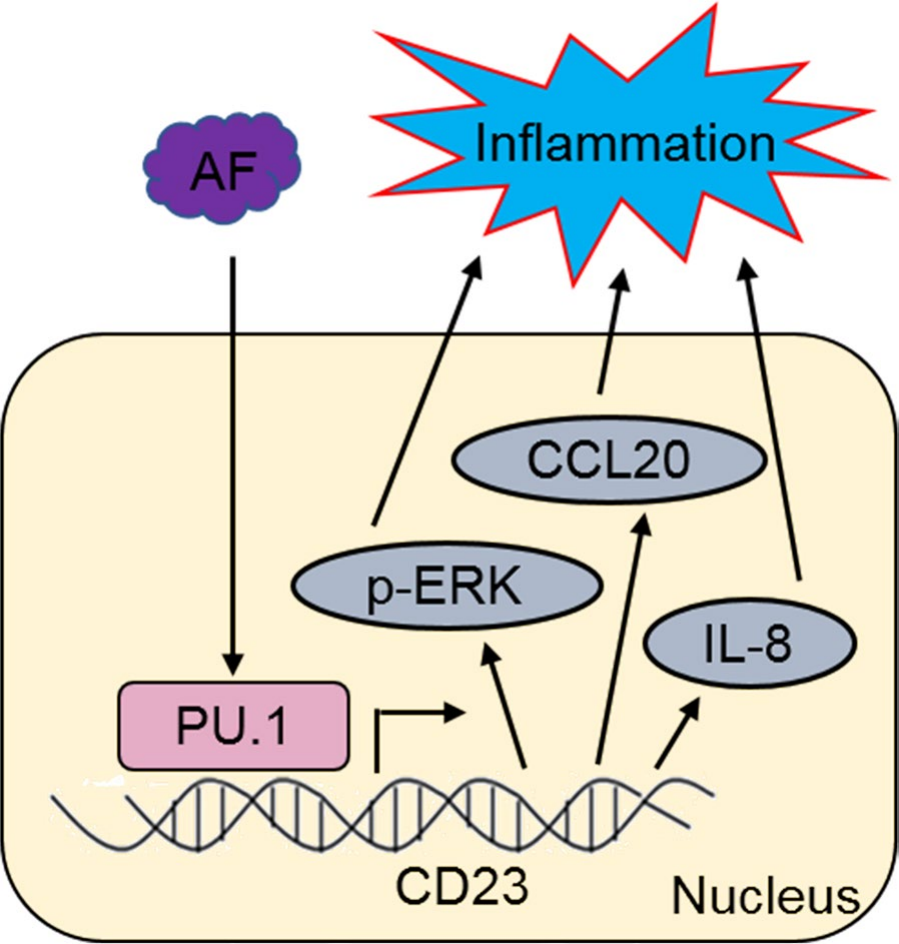
A schematic depicting the PU.1-CD23 axis regulating inflammation response under AF conditions

It is widely regarded that crucial inflammatory factors released by immune cells play important roles in host antifungal immunity [39]. Inflammations involving immunity are normal responses to infection [40]. In this study, the expressions of p-ERK and the pro-inflammatory factors CCL20, IL-8, IL-1β, IL-6, TNF-α and IL-12 enhanced with AF infection in HTMs. In PU.1-OE HTMs, the expression levels of the pro-inflammatory factors CCL20, IL-8, IL-1β, IL-6, TNF-α and IL-12 were significantly elevated. Furthermore, the overexpression of CD23 in the PU.1 siRNA groups significantly increased the expressions of the inflammatory factors IL-1β, IL-6, TNF-α and IL-12 that were reduced with PU.1 silencing under AF conditions. Thus, PU.1-CD23 signaling exerted crucial roles on driving AF-mediated inflammatory response (Fig. 7). Liu et al. reported that overexpression of PU.1 promoted the release of pro-inflammatory cytokines, and enhanced the phagocytosis and killing ability of THP-1-derived macrophages during AF stimulation [38]. Manjula et al. reported that decreased inflammatory cytokine release and survival benefits were observed in mice with macrophages that lacked functional PU.1, which were challenged with lipopolysaccharide, a component of the outer membrane of gram-negative bacteria [41]. Efficient protection against fungal infection requires a fine-tuned balance between pro-inflammatory cytokines and anti-inflammatory cytokines. Thus, PU.1 may be an essential factor for defence against AF infection, and overexpression of PU.1 may be a novel method for the prophylaxis or treatment of invasive pulmonary aspergillosis.

Previous research indicated that the signaling activated by PRRs led to the expressions of pro-inflammatory cytokines to recruit immune cells, establishing the "antiviral state" [42]. And the inflammatory response represents a coordinated set of physiologic actions that serve to fight infection, and promote recovery from external stressors [43]. In this study, the histological results from HE staining proved that exogenous PU.1 and CD23 by adenovirus transfection attenuates pathological defects and inflammatory lesions in immunodeficient mouse lung tissues with AF infection, which suggested that PU.1 and CD23 were irreplaceable components exerting critical roles in human innate immunity dampening Aspergillus-triggered immunopathology.

## Conclusions

In conclusion, this study elucidated that the PU.1-CD23 signaling pathway mediated innate immunity against AF infection in HTMs and mice by driving inflammatory response. Although more investigations are needed in future studies, with the deepening of gene therapy and its wide clinical application, PU.1/CD23 may become a new anti-aspergillosis therapeutic for the prevention and treatment of invasive aspergillosis.

## Supplementary Information

**Additional file 1**. The full length original gel blot images.

**Additional file 2. Fig. S1**. CD23 overexpression upregulated the expressions of inflammation factors IL-1β, IL-6, TNF-α and IL-12 that downregulated with PU.1 interference.

## Data Availability

The datasets used and/or analysed during the current study are available from the corresponding author on reasonable request.

## Abbreviations

AF: *Aspergillus fumigatus*
AMs: Alveolar macrophages
HTMs: Human THP-1-derived macrophages
IA: Invasive aspergillosis
PRR: Pattern-recognition receptor
TLRs: Toll-like receptors
CLRs: C-type lectin receptors
NLRs: Nod-like receptors
RLRs: RIG-I like receptors
CRDs: C-type lectin domains
ETS: E26 transformation-specific
CD23: differentiation antigen 23
FCER2: Fc fragment of the IgE receptor II
p-ERK: Phosphorylated-extracellular signal-regulated kinase
CCL20: Chemokine ligand 20
