# Supplementary material for: PU.1-CD23 signaling mediates pulmonary innate immunity against Aspergillus fumigatus infection by driving inflammatory response
Source: BMC Immunol. 2023 Jan 17;24:4. doi: 10.1186/s12865-023-00539-2 (PMC9844028; doi:10.1186/s12865-023-00539-2)
Supplement: Supplementary file 1 — Additional file 1. The full length original gel blot images. [file 12865_2023_539_MOESM1_ESM.docx]

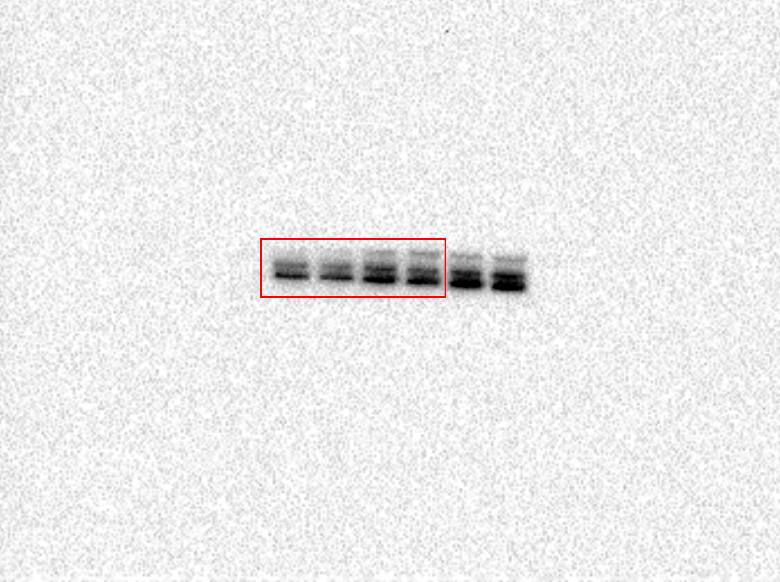


Figure 1E (PU.1)

Time of exposure: 20.8 second, but marker didn't take a picture.


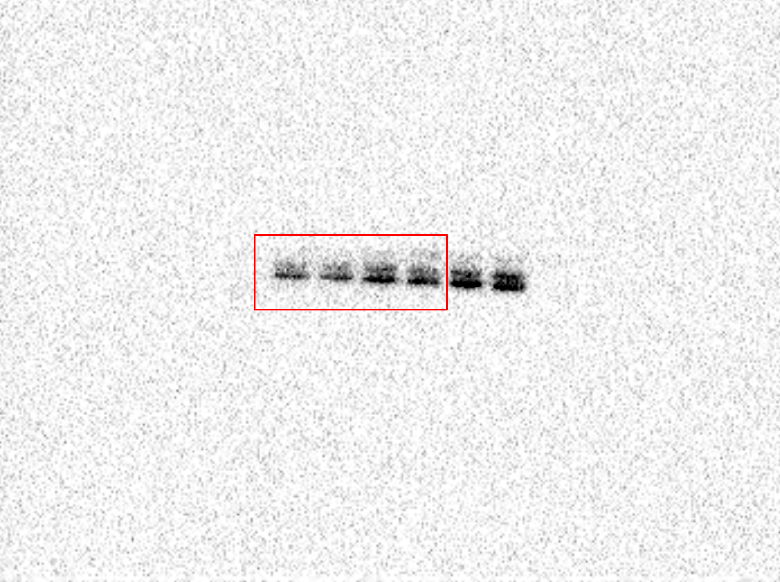


Figure 1E (PU.1)

Time of exposure: 1 second, but marker didn't take a picture.


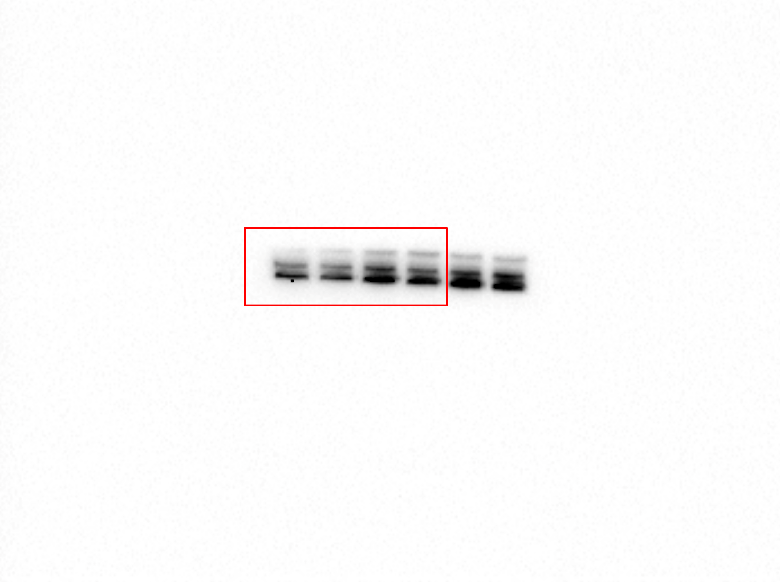


Figure 1E (PU.1)

Time of exposure: 46.1 second, but marker didn't take a picture.


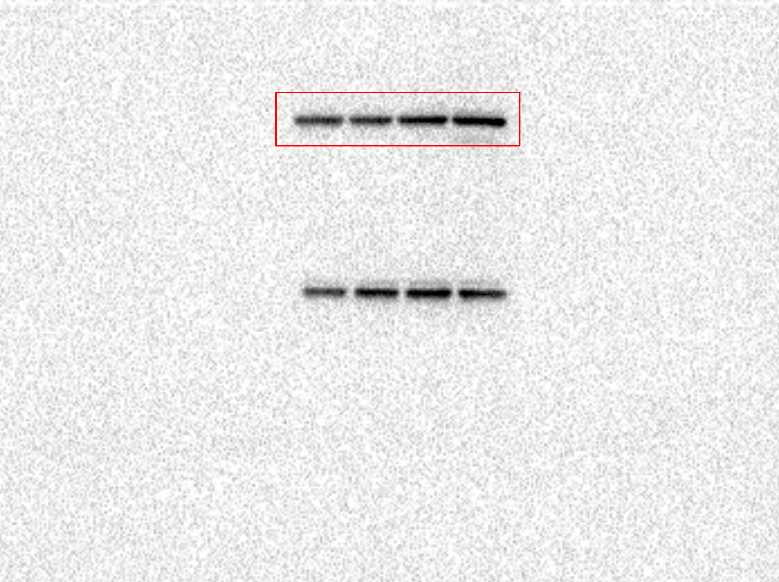


Figure 1E (CD23)

Time of exposure: 2.3 second, but marker didn't take a picture.


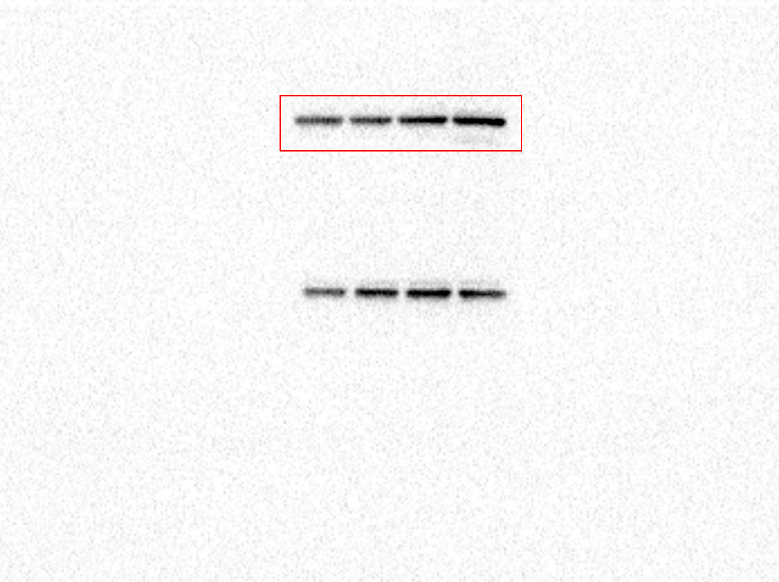


Figure 1E (CD23)

Time of exposure: 1 second, but marker didn't take a picture.


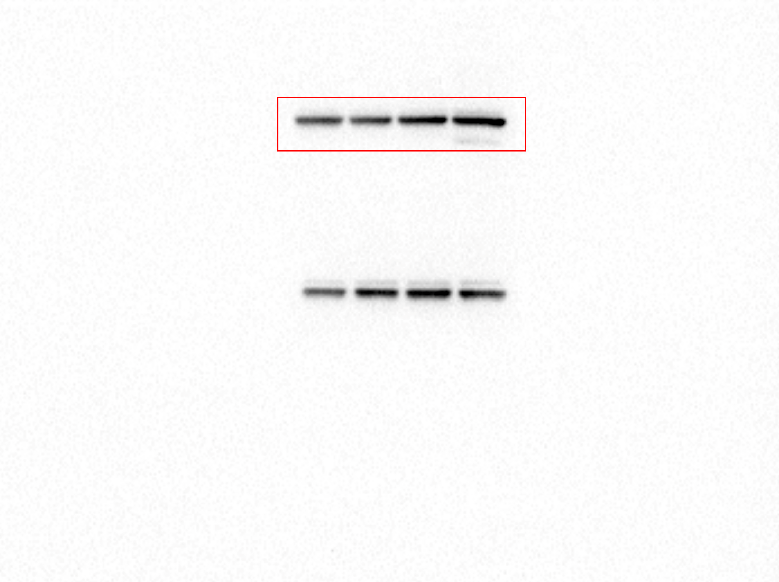


Figure 1E (CD23)

Time of exposure: 5.7 second, but marker didn't take a picture.


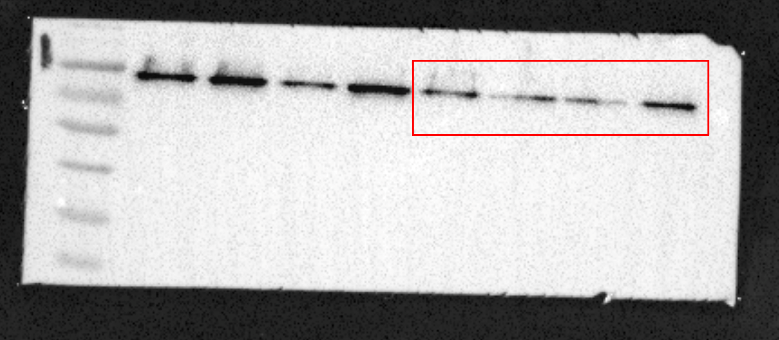


Figure 1E (p-ERK)


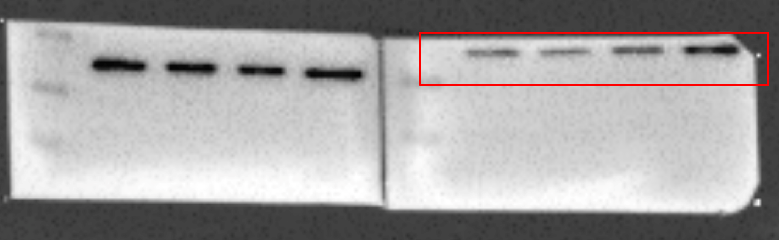


Figure 1E (CCL20)


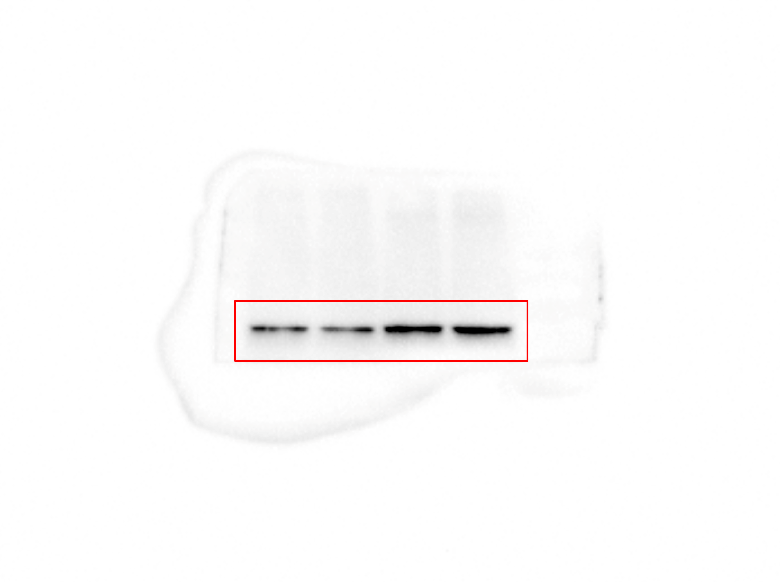


Figure 1E (IL-8)

Time of exposure: 2.3 second, but marker didn't take a picture.


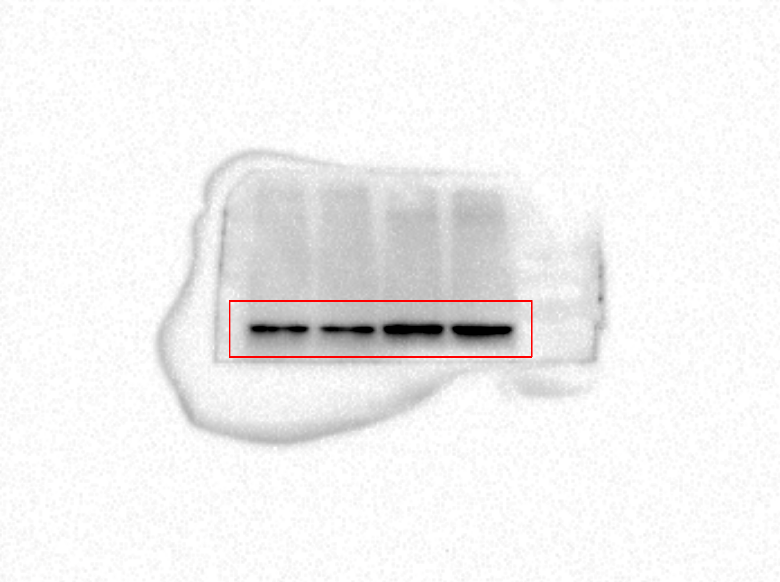


Figure 1E (IL-8)

Time of exposure: 15.9 second, but marker didn't take a picture.


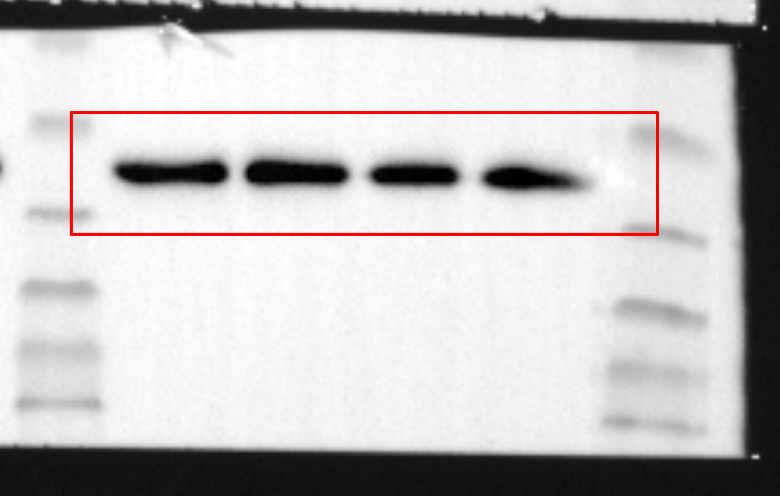


Figure 1E (GAPDH)


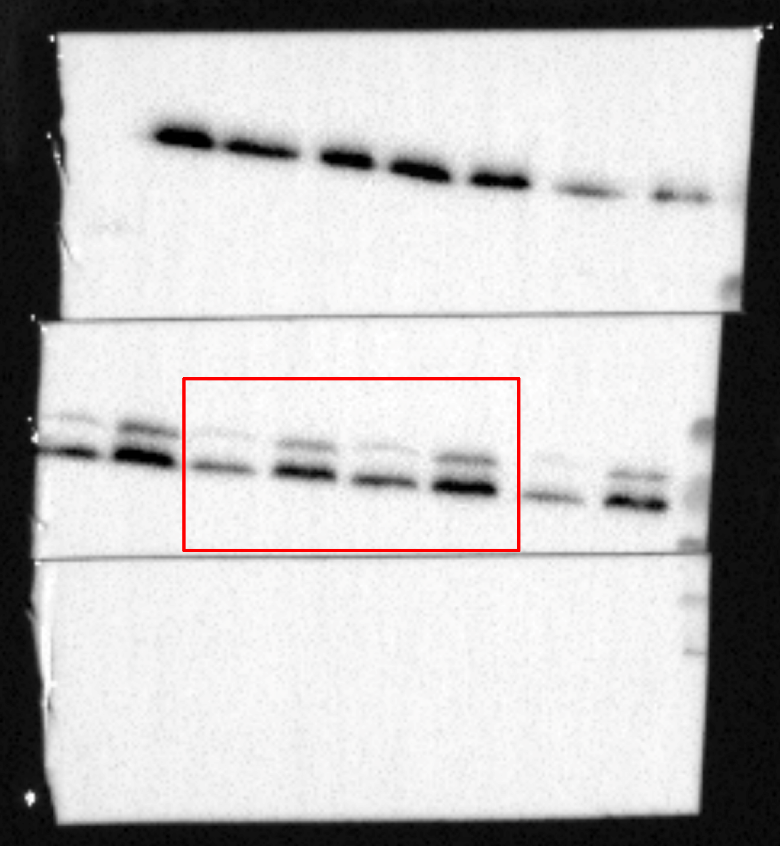


Figure 6D (PU.1)


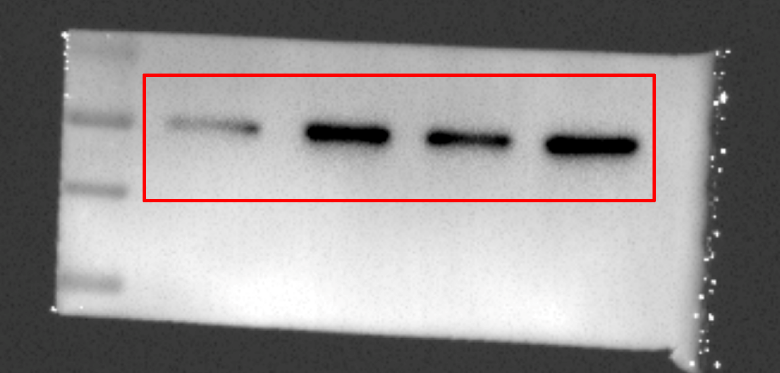


Figure 6D (CD23)


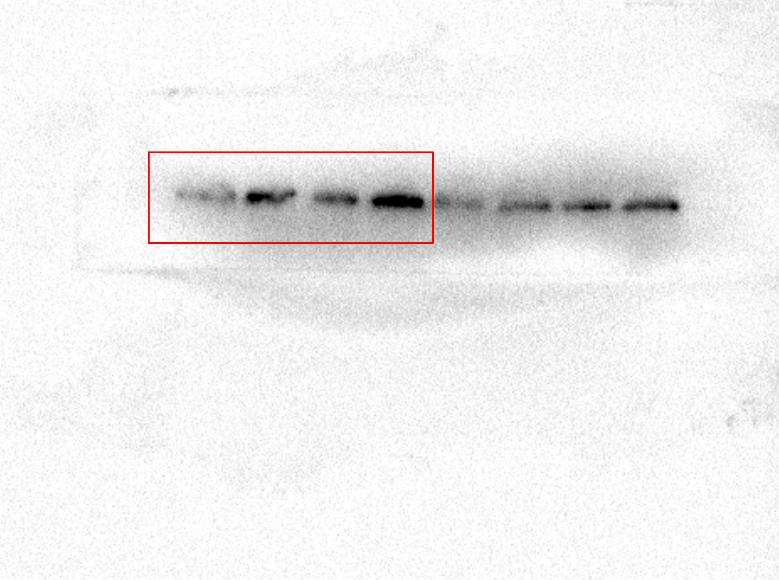


Figure 6D (p-ERK)


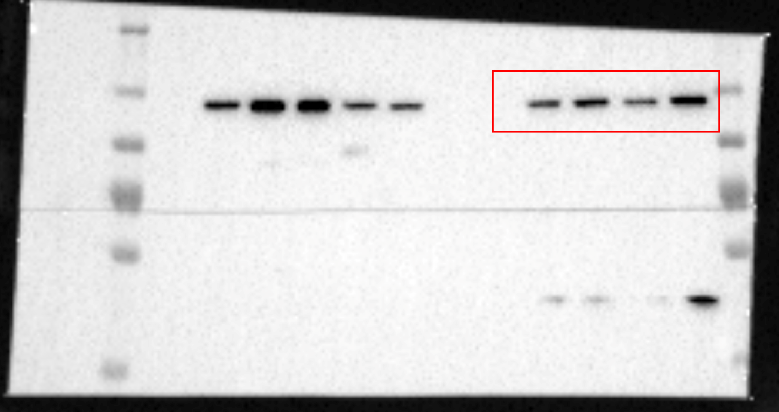


Figure 6D (CCL20)





Figure 6D (IL-8)





Figure 6D (GAPDH)

Time of exposure: 1 second, but marker didn't take a picture.





Figure 6D (GAPDH)

Time of exposure: 10 second, but marker didn't take a picture.


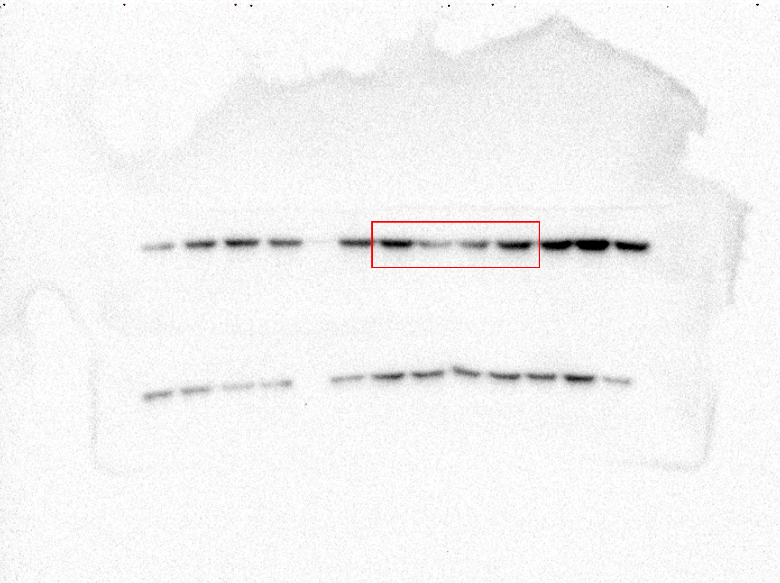


Supplemental Figure 1A (CD23)


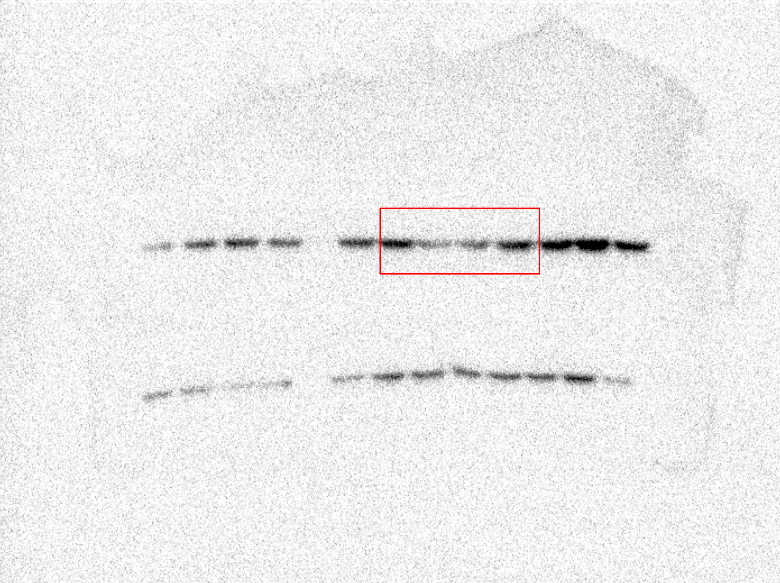


Supplemental Figure 1A (CD23)

Time of exposure: 1 second, but marker didn't take a picture.


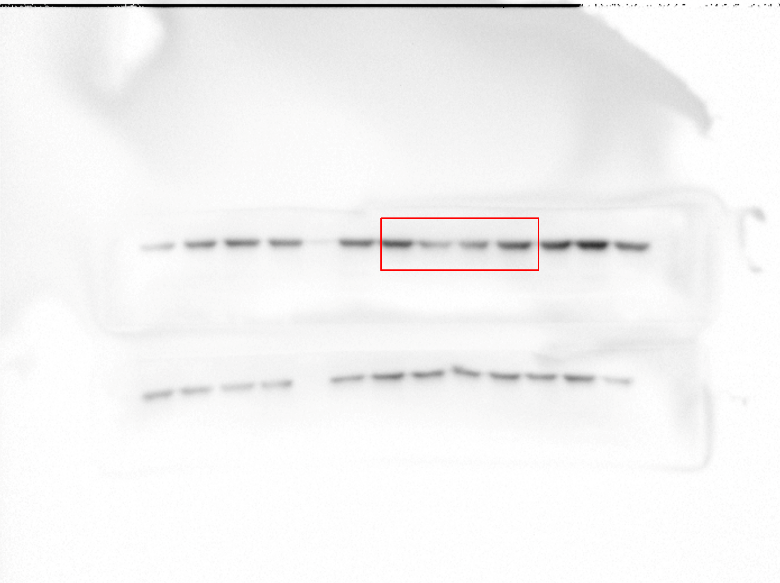


Supplemental Figure 1A (CD23)

Time of exposure: 60 second, but marker didn't take a picture.


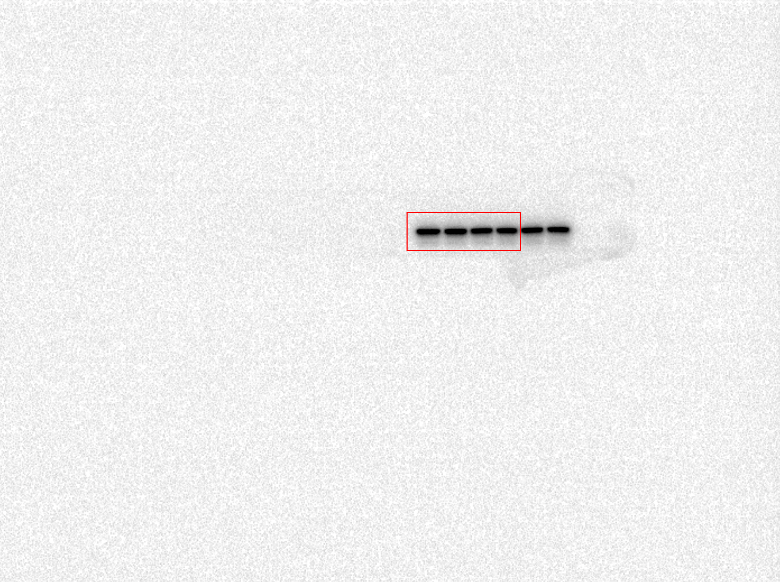


Supplemental Figure 1A (GAPDH)

Time of exposure: 1 second, but marker didn't take a picture.


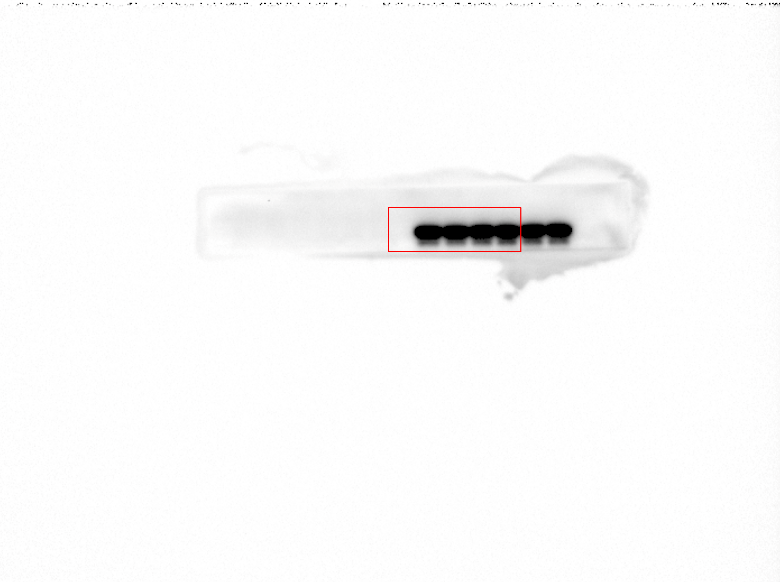


Supplemental Figure 1A (GAPDH)

Time of exposure: 58.4 second, but marker didn't take a picture.


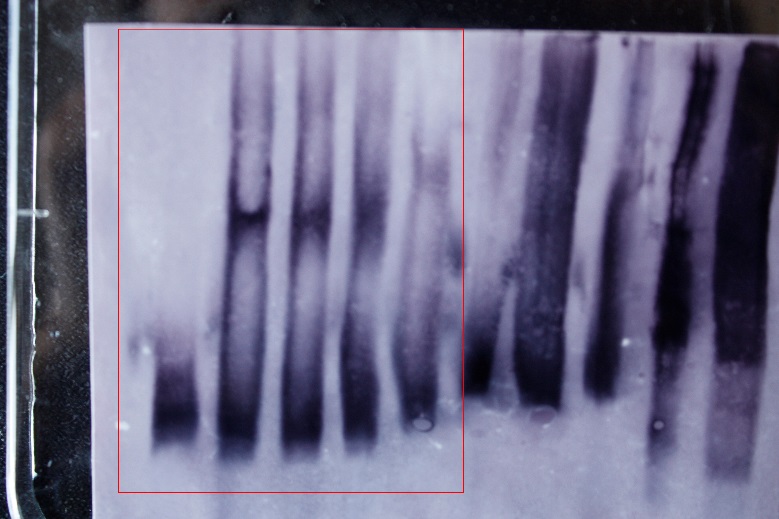


Figure 4H
