# Supplementary material for: PU.1-CD23 signaling mediates pulmonary innate immunity against Aspergillus fumigatus infection by driving inflammatory response
Source: BMC Immunol. 2023 Jan 17;24:4. doi: 10.1186/s12865-023-00539-2 (PMC9844028; doi:10.1186/s12865-023-00539-2)
Supplement: Supplementary file 2 — Additional file 2. Fig. S1. CD23 overexpression upregulated the expressions of inflammation factors IL-1β, IL-6, TNF-α and IL-12 that downregulated with PU.1 interference. [file 12865_2023_539_MOESM2_ESM.docx]

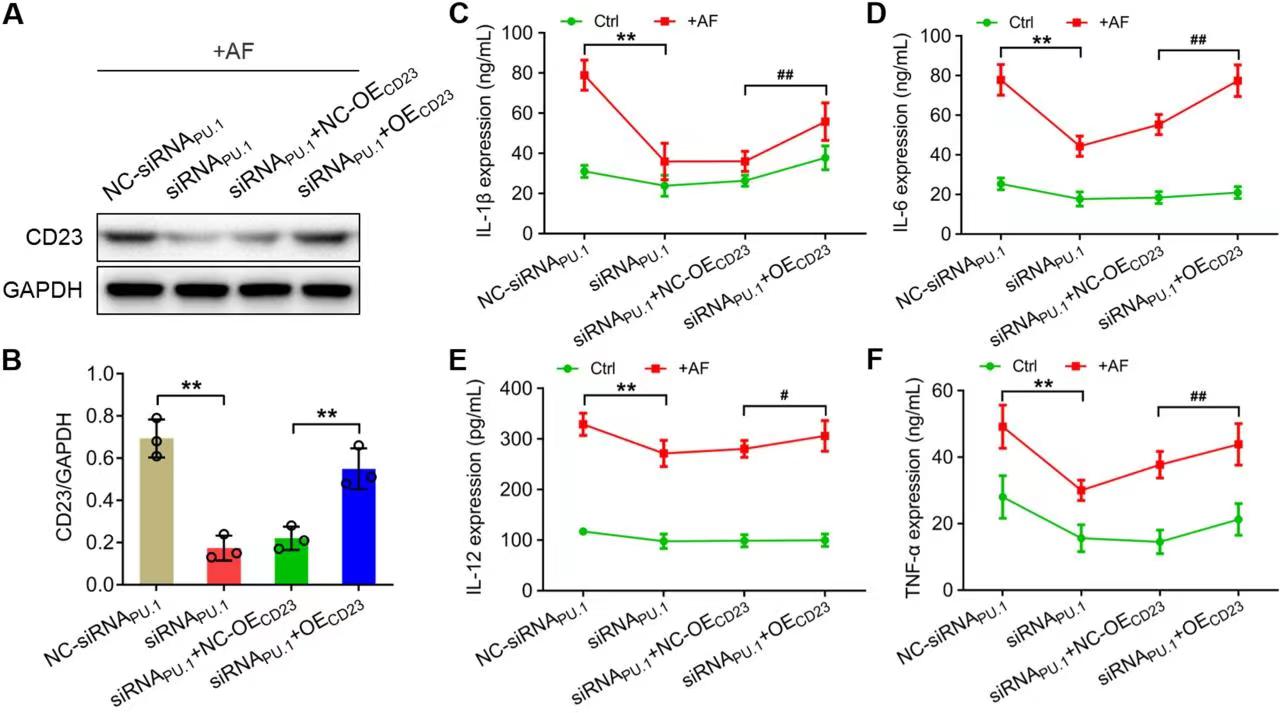


**Supplemental Fig. 1** CD23 overexpression upregulated the expressions of inflammation factors IL-1β, IL-6, TNF-α and IL-12 that downregulated with PU.1 interference. **A** Western blotting analysis showed the expression of CD23 and in HTMs with AF infection with siRNA PU.1 and siRNA PU.1 + OECD23 normalized to GAPDH. The samples derived from the same experiment and that gels/blots were processed in parallel. **B** Quantitative analysis of the relative protein level of CD23 in A. **C-F** Expression of the inflammatory factors IL-1β (C), IL-6 (D), IL-12 (E) and TNF-α (F) in siRNA PU.1 and siRNA PU.1 + OECD23 HTMs with AF infection. All data are presented as the mean ± SD, N ≥ 3, **P* < *0.05*, ***P* < *0.01*.
